# The role of executives’ social capital in improving merger and acquisition performance during corporate transformation and upgrading: Evidence from Chinese media enterprises

**DOI:** 10.1371/journal.pone.0306363

**Published:** 2024-09-23

**Authors:** Wancheng Yang, Qi Zeng

**Affiliations:** 1 School of Logistics and e-Commerce, Zhejiang Wanli University, Ningbo, China; 2 Key Research Institute of Philosophy and Social Sciences of Zhejiang Province–Modern Port Service Industry and Creative Culture Research Center, Ningbo, China; 3 International Exchange College, Zhejiang Business Technology Institute, Ningbo, China; Federal University of Goias: Universidade Federal de Goias, BRAZIL

## Abstract

During the transformation and upgrading of enterprises, executives’ social capital provides useful access to resources through merger and acquisition (M&A) strategies. This study examines 145 M&A events of Chinese listed media enterprises undergoing transformation and upgrading as research samples. It empirically analyzed the impact of executives’ social capital on short-term and long-term M&A performance from three aspects: corporate social capital (CSC), political social capital (PSC), and financial social capital (FSC). It also confirmed the moderating effect of corporate ownership structure, exploring the mechanism of executives’ social capital during the period of transformation and upgrading. Based on the empirical results, we found that: (1) CSC significantly enhances short-term M&A performance but has no significant effect on long-term performance; (2) PSC positively influences both short-term and long-term M&A performance. State-owned media enterprises may gain relatively fewer benefits from PSC in the short term after M&A, but they can accrue more significant benefits in the long term post-M&A; (3) FSC does not affect short-term M&A performance but exerts a negative impact on long-term performance. The negative effect is even more pronounced in state-owned enterprises. This study complements existing research on executives’ social capital during the transformation and upgrading of enterprises. It provides a reference for media enterprises in China and other emerging economies to utilize executives’ social capital.

## 1. Introduction

Under the influence of various factors such as market competition pressure and macroeconomic policy guidance, the transformation and upgrading of enterprises have gradually become a key focus for both the industry and academia. In this context, "endogenous growth" and "external expansion" have been identified as the dual strategies for enterprises to achieve transformation and upgrading [1] (Zhu & Li, 2018). "External expansion" strategy primarily involves acquiring external resources through mergers and acquisitions (M&A) to facilitate the enterprise’s transformation and upgrading [2]. This strategy is characterized by the rapid expansion of business scale and swift achievement of strategic transformation, making it a critical pathway for enterprises to quickly achieve transformation and upgrading, thereby enhancing their competitive advantage amid significant market competition pressure [3]. However, M&A, as a complex economic activity, not only promotes the transformation and upgrading of enterprises but also introduces significant risks [4]. Therefore, improving M&A performance during the transformation and upgrading process has become a major focus for enterprises.

According to resource dependence theory, the ability of enterprise executives to interact with and acquire resources from the external environment can significantly affect the enterprise’s M&A behavior and performance [5, 6]. Additionally, network embeddedness theory suggests that executives’ strategic decisions and their implementation during M&A activities are influenced by their social capital [7, 8]. Building upon this premise, numerous scholars have integrated resource dependence theory, social capital theory, and network embeddedness theory to examine how executives’ social capital affects M&A performance [9–11]. Lin (2002) defined social capital as resources embedded within social networks [12], highlighting that the amount of executives’ social capital is directly related to the size and strength of their social networks [13, 14]. Consequently, most existing studies use social network analysis to quantitatively assess the impact of executives’ social capital on M&A performance from a sociological standpoint [15, 16]. In the context of China’s relational society [17], the social capital derived from various relationships has increasingly become a focus of research, highlighting the diverse impacts of different resources. For instance, Pan et al. (2008) examined the relationship between executives and the government (i.e., political connections) in their research on M&A performance [18]. They found that political connections could mitigate the negative influence of government interventions. Huang & Zhu (2014) examined the influence of executives’ financial connections [19], discovering that while these affiliations could encourage the occurrence of M&A, they did not enhance post-M&A operational performance. Further research has expanded beyond the impact of specific types of relational resources on M&A to include a wider array of social relationships within the framework of social capital. Wang et al. (2012) identified executives’ internal industry connections and political connections as social capital [20], arguing that social capital, as an informal institution, can make up for the deficiencies of formal institutions such as the market environment and government intervention. Building on this, Qi & He (2015) broadened the concept of executives’ social capital to include internal industry connections, political connections, and financial connections [21], noting that social capital within financial and governmental networks positively influences the value effects of M&A.

There is no consensus in existing research regarding the influence of executives’ social capital on M&A performance. As a result, scholars have started to explore the specific effects of executives’ social capital in various developmental contexts, such as uncertain business environments [22], specific industries [23], family-owned enterprises [24], and within the framework of globalization [25]. However, the specific influence of executives’ social capital on M&A performance during transformation and upgrading remains to be thoroughly investigated. Amid the continuous evolution of China’s policy landscape for the media industry, driven by capital and technological advancements, the sector has undergone a comprehensive industrial transformation [26]. Despite this, the rapid rise of Internet-related sectors has led to a swift decline in revenues among Chinese media enterprises [27]. In response to national policy directives, Chinese media enterprises have embarked on a new phase of transformation and upgrading, aiming to solidify their competitive edge within this evolving developmental milieu [28]. During this phase, numerous Chinese media enterprises have rapidly enhanced their competitive standing by integrating emerging technology enterprises specializing in digital video, gaming, and marketing [29]. The large-scale M&A within China’s media industry provide a robust empirical foundation for investigating corporate M&A behavior in the process of transformation and upgrading. This study examines M&A activity in the context of enterprise transformation and upgrading and explores the impact of executives’ social capital on M&A performance, thereby providing invaluable insights for Enterprises in China and other developing economies undergoing similar transformation and upgrading, and complementing existing theories on enterprise transformation and upgrading, as well as mergers and acquisitions.

The contributions of this study are as follows: First, while many studies have explored issues in the process of enterprise transformation and upgrading from a macro perspective, such as employee participation [30] and key areas of transformation [31], existing research rarely examines M&A in the developmental context of transformation and upgrading and identifies the key factors that enhance M&A performance. In view of that, this study has built a theoretical framework by integrating network theory, resource dependence theory, and institutional theory, and used Chinese media enterprises undergoing transformation and upgrading as an example to explore whether executives’ social capital affects M&A performance, thereby complementing existing theories on enterprise transformation and upgrading. Second, existing research on M&A has not reached a unified conclusion on the influence of executives’ social capital. This inconsistency is caused by the varying developmental contexts of the M&A samples. Under the guidance of top-level policies, Chinese media enterprises have embarked on large-scale transformation and upgrading, providing a strong foundation for studying M&A in this context. This study places M&A within the context of transformation and upgrading, and clarifies the specific role of executives’ social capital in this developmental context, thereby contributing to refining the mechanism of how executives’ social capital influences M&A performance. Finally, this study subdivides executives’ social capital into corporate social capital, political social capital, and financial social capital. It not only explores the direct impact of executives’ social capital on M&A performance but also examines the moderating effect of different institutional environments on this impact. Additionally, it investigates the evolution of the impact of executives’ social capital, thereby supplementing the research on heterogeneous social capital in M&A-related theories.

The rest of this study is organized as follows: Section 2 reviews relevant literature and proposes research hypotheses. Section 3 describes the sample data source, research models, and variables. Section 4 presents the results of the empirical analysis. The empirical results are discussed in Section 5, and Section 6 is a summary of the study.

## 2. Construction of theoretical framework

### 2.1 Concept of social capital

Currently, scholars have defined the concept of executives’ social capital from various perspectives, including relational networks [32, 33], social reputation [34], resource advantages [35, 36], and personal abilities [37]. These concepts all relate executives’ social capital to the resources or capabilities they can utilize [38]. According to Lin’s (2002) description of social capital [12], executives’ social capital is embedded in their social structure and can be obtained or mobilized through purposeful actions. In China, social capital is generally referred to as "guanxi" [3], and executives’ social capital typically originates from personal relationships such as colleagues and friends [39]. Therefore, from the perspective of network theory, this study defines executives’ social capital as resources embedded within their social networks.

In China, different relational networks bring different relational resources, resulting in the heterogeneity of social capital [40]. Within executives’ relational networks, different members often possess different types of resources [41]. Thus, from the perspective of network theory, this study subdivides executives’ social capital into corporate social capital, financial social capital, and political social capital [42]. Specifically, corporate social capital refers to the resources contained within executives’ networks with other enterprises [43]; financial social capital refers to the resources contained within executives’ networks with financial institutions [44]; and political social capital refers to the resources contained within executives’ networks with government departments [45, 46].

### 2.2 Theoretical framework

In this study, a theoretical framework is built by integrating network theory, resource-based theory, and institutional theory to discuss the relationship between executives’ social capital and M&A performance, as well as whether different institutional environments of enterprises moderate this relationship. According to network theory and resource-based theory, the social capital embedded in the various relational networks of executives can provide critical resources and information for M&A strategies. Additionally, executives can use their networks to access non-public market information, reducing search and negotiation costs, and thereby gaining a competitive advantage in M&A activities [47]. Therefore, executives’ social capital not only provides essential information for M&A decision-making but also plays a crucial role in implementing the decision.

**Fig 1:**
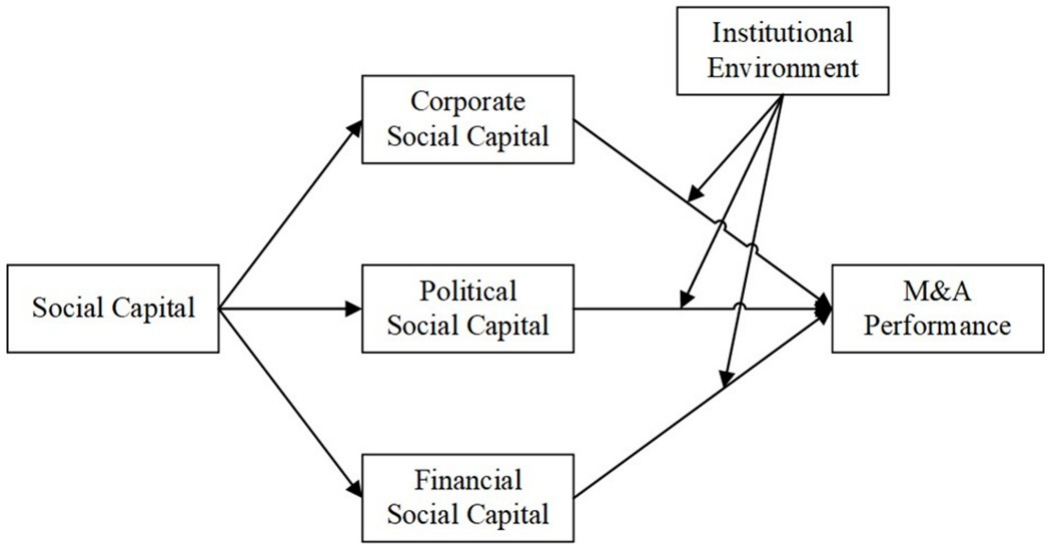
Theoretical framework.

Although network theory and resource-based theory offer strong theoretical support for understanding the impact of social capital on M&A performance, the differences in this impact under different institutional contexts deserve attention. Chinese media enterprises can be divided into state-owned enterprises (SOEs) and private enterprises based on ownership. State-owned media enterprises emerged from media public institutions through the "enterprise transformation and restructuring" process, which involved converting parts of public institutions with market potential and operational characteristics into market-oriented entities, thereby forming enterprises capable of operating independently under market economy conditions [48]. While this restructuring prompted these enterprises to make corresponding adjustments and reforms in their management systems and operational mechanisms, the "dual attributes" of Chinese media enterprises mean that state-owned media enterprises maintain strong connections with their original public institutions and supervisory government departments [49], for example, executives are still appointed by supervisory government departments. This leads to a significant institutional difference compared to private media enterprises. Therefore, this study employs institutional theory to explore how the institutional differences between state-owned and private media enterprises moderate the relationship between social capital and M&A performance. The theoretical framework of this study is shown in Fig 1.

### 2.3 Research hypotheses

#### (1) The impact of CSC on M&A performance

Executives’ corporate social capital (CSC) consists of various resources embedded in their networks with other enterprises [50]. The amount of CSC is related to the executives’ position within the corporate network; for example, executives situated at the network’s center often possess unique informational advantages [51]. Utilizing corporate networks as channels, executives can significantly enhance their ability to acquire knowledge, perspectives, or information, which are important for corporate M&A [52, 53]. Specifically, the information and experiential knowledge that executives obtain from corporate networks can effectively reduce uncertainties involved in M&A target identification and valuations [54–56]. During target identification, information resources from the corporate network can help executives identify M&A opportunities, thereby reducing the search costs for potential targets [57]. During target valuation, due to factors such as information asymmetry, targets may be overvalued, leading to high acquisition premiums [58]. By leveraging corporate networks, executives can access information about M&A targets and their industries, thereby reducing information asymmetry between the involved parties [16]. Furthermore, executives’ corporate social capital can effectively reduce uncertainties during the post-M&A integration phase [59, 60]. For example, executives are more likely to acquire similar M&A case studies through their corporate networks and use them as references for resolving related issues [61, 62]. Based on this, we propose Hypothesis 1.

Hypothesis 1: CSC has a positive impact on M&A performance.

#### (2) The impact of PSC on M&A performance

In the development of the world economy, it is common for enterprises to establish political connections with governments [63]. Executives’ political social capital (PSC) exists within their connections with governmental entities [64]. For enterprises, the government can show an "exploitative hand" when it uses corporate M&A to address local fiscal deficits, and employment issues, or achieve political goals [65], or extend a "helping hand" when it employs M&A to support local failing enterprises [66]. In China, the media industry has transitioned from "public institutions" to "enterprise institutions," but the government still retains control over key resources such as media editorial departments [67]. Under policy directives such as media integration, the government and market have driven continuous reform and development of China’s media industry through their joint efforts [68, 69]. Additionally, since China’s market allocation mechanisms are not yet fully mature [70], the government still controls key resources critical to enterprise development, such as credit allocation by state-owned banks, government subsidies, and tax incentives [71]. For instance, government-led financial investments and industrial funds play an important role in the development of Chinese media Enterprises [72]. Although the government has the ability and motivation to show an "exploitative hand" in corporate M&A, it has shown more of a "helping hand" towards the development of listed media Enterprises [73]. Based on this, we propose Hypothesis 2.

Hypothesis 2: PSC has a positive impact on M&A performance.

#### (3) The impact of FSC on M&A performance

Executives’ financial social capital (FSC) exists within their connections with financial institutions [74] and influences the economic activities and outcomes of an enterprise. Ciamarra (2006) found that when an enterprise’s executives have a banking background, it can have greater access to loans, thereby reducing financing costs [75]. Financial support is crucial for enterprises undergoing transformation and upgrading, whether through “endogenous growth” or “external M&A”. Therefore, executives’ FSC provides a solid foundation for corporate financing [19]. In addition to financial support, financial institutions also have advantages in professional knowledge and information resources [76, 77]. Executives’ FSC contains valuable business information, which can reduce information asymmetry and decrease costs involved in M&A [2], thereby improving M&A performance. Based on this, we propose Hypothesis 3.

Hypothesis 3: FSC has a positive impact on M&A performance.

#### (4) Moderating effect of corporate property right

As the Chinese media market opens up, an increasing number of private enterprises are entering the media industry and undergoing transformation and upgrading amid the trend of media integration [78]. However, compared with state-owned media enterprises, private enterprises face significantly different institutional environments. In terms of corporate social capital (CSC), executives of state-owned enterprises have fewer opportunities to hold positions in other organizations due to stricter position management and supervision. In contrast, executives of private enterprises are often more flexible and autonomous, making it easier for them to hold positions in different organizations [79]. This means that executives of private media enterprises may hold more positions in other organizations, and therefore have access to more information in their CSC [80]. Nonetheless, more positions may lead to busy multitasking [81], thereby reducing executives’ efficiency and regulatory effectiveness in M&A. In terms of political social capital (PSC), executives of state-owned enterprises typically have more associations with the government due to the personnel appointment system [82], therefore, they tend to have greater access to key resources in their PSC compared to executives in private enterprises. In China, having abundant government social capital means that enterprises are more likely to receive support from banks and government finances during the transformation and upgrading process [83]. Although the Chinese media market has significantly opened up since the implementation of the reform and opening-up policy, key media resources are still under government control [67]. Therefore, private media enterprises actively seek political connections to gain rent-seeking interest, although such connections may bring potential political and social burdens [84]. Regarding financial social capital, most financial resources are concentrated in state-owned enterprises in China [85], which means private enterprises face more financing constraints in their development [86]. Although privately listed media enterprises can mitigate financing constraints by hiring executives with backgrounds in financial institutions, financing issues still exist [87]. From the perspective of institutional theory, the influence of executives’ social capital on M&A performance may vary due to differences in the institutional environments of state-owned and private media enterprises. Based on this, we propose Hypothesis 4.

Hypothesis 4: Corporate property rights can moderate the impact of social capital on M&A performance.

## 3. Methodology

### 3.1 Data collection and processing

In this study, we considered two factors when deciding the time frame for collecting M&A event samples among Chinese listed media Enterprises. First, China implemented new accounting standards in 2007, and our model requires financial indicators from the year before the M&A announcement. Therefore, we set January 2008 as the starting point for collecting M&A event samples. Second, the Chinese market suffered a severe impact from the COVID-19 pandemic in 2020, and to avoid its influence on M&A performance two years post-M&A, we chose December 2017 as the endpoint for collecting M&A event samples. Based on this, we collected all M&A events involving Chinese listed media enterprises from January 2008 to December 2017 using the CSMAR database. Notably, these media enterprises were all listed on the Shanghai and Shenzhen stock exchanges before 2017, totaling 118. While reviewing the M&A event information, we found issues such as undisclosed specific transaction times, consecutive M&As, and unexpected termination of M&A. Therefore, it was necessary to clean the M&A events to obtain a sample set with minimal noise interference. To that end, we established the following cleaning rules.

(1) removing M&A events where the specific announcement date or transaction date is not available, as the event time window is not clear. (2) removing M&A events where the acquirer is not a listed media enterprise. (3) removing M&A events involving media enterprises whose stocks have been specially treated (ST), as these events may be influenced by factors related to ST status. (4) removing M&A events where the target enterprises ’s registered location is not within mainland China, due to the unique nature of cross-border M&A which may cause deviations from other M&A events. (5) removing M&A events where the acquisition funds are less than 5 million yuan, the acquisition shares are less than 5%, or the acquisition does not change the controlling position of the acquiring enterprises, as these small-scale M&A events have no significant impact on the stock price of the acquiring enterprises. (6) removing M&A events where the acquisition announcement was made but the M&A contract was eventually terminated, or where the acquirer no longer holds shares in the target enterprises during the observation period. (7) If multiple M&A events are initiated by a company on the same event day, and the same target equity is acquired from different shareholders, merge these M&A events into a single event. (8) If multiple M&A events are initiated by an enterprise on the same event day, and the targets are different, select the M&A event with the largest transaction amount. (9) If a listed media enterprise conducts consecutive M&A events on multiple event days, this study, referencing the methods used by Zhu Tao (2006) and He Ren (2014), retains only the first M&A event within three months [88, 89]. After cleaning the data according to the above rules, we finally obtained 145 valid M&A transactions involving listed media enterprises as the research sample.

### 3.2 Research model

#### 3.2.1 Measurement of M&A performance

In this study, we use M&A performance as the dependent variable and divide it into short-term performance (SP) and long-term performance (LP).

*(1) Short-term performance*. The short-term performance is measured using the Cumulative Abnormal Returns (CAR) calculated through an event study based on the market model. Following the common practice among scholars, as referenced from Li & Zhu (2005) and Chen (2013), we estimate the intercept term *α*_*i*_ and the systematic risk coefficient *β*_*i*_ in market model regression equation *R*_*it*_ = *α*_*i*_*+β*_*i*_*R*_*mt*_ *+ ε*_*it*_ for the estimation window, which spans from 180 trading days before the M&A announcement to 30 trading days before the announcement. In the market model, *R*_*it*_ represents the stock return of company i at time t, and *R*_*mt*_ represents the average market return of the corresponding stock market. Abnormal returns, denoted as *AR*_*it*_, are calculated based on the formula ${AR}_{it}=R_{it}-\left(\alpha_{i}^{'}+\beta_{i}^{'}R_{mt}\right)$. We select the [−10,10] window as the event window for analysis, and the CAR is obtained by aggregating the abnormal returns *AR*_*it*_ within this window.

*(2) Long-term performance*. Long-term performance is measured by calculating the Buy-and-Hold Abnormal Returns (BHAR) for 24 months after the merger or acquisition of the listed media company. This measurement is carried out using a paired sample combination method. Specifically, Enterprises listed on the stock exchange are sorted into five equally-sized groups based on their market capitalization as of June 30th of the merger year, from smallest to largest. They are then further divided into five groups based on the ratio of earnings per share for the year preceding the merger to the year-end closing price for the merger year, again sorted from smallest to largest. By combining these groups in a cross-manner, pairs of sample combinations are created. The average monthly return for the t-th month of these paired sample combinations is regarded as the expected return (*R*_*pt*_) for the corresponding company in the t-th month. The calculation of long-term performance during the observation window is represented by ${BHAR}_{24}=\prod_{t=1}^{24}\left(1+R_{t}\right)-\prod_{t=1}^{24}\left(1+R_{pt}\right)$, where *R*_*t*_ denotes the stock return for the t-th month.

#### 3.2.2 Measurement of executives’ social capital

From the perspective of relational networks, the characteristics of executives’ social networks (such as network size, member characteristics, and relationship types) can indirectly determine the characteristics of their social capital [90]. Based on this, we divide social capital (SC) into corporate social capital, political social capital, and financial social capital according to member characteristics.

We constructed a corporate relational network based on the positions held by executives in other companies and used network centrality to measure CSC [50]. In this network, eigenvector centrality is used to measure an actor’s position within the network [91]. Actors with higher positions can acquire more social capital within the relational network [8]. Eigenvector centrality is used to measure CSC in this study. The formula for calculating eigenvector centrality is as follows:

AX=λX (1)

$$\lambda_{i}x_{i}=a_{1i}x_{i}+a_{2i}x_{i}+\cdots +a_{it}x_{i}+\cdots +a_{ni}x_{n},\;(i\neq t)$$ (2)

$$C_{(e)i}=\lambda_{i}$$ (3)

In the calculation formula for eigenvector centrality, *A* represents an *n* × *n* adjacency matrix composed of *a*_*ij*_; *X* = (*x*_1_,*x*_2_,*x*_3_,⋯,*x*_*n*_)^T^ represents the degree centrality of each node; *λ*_*i*_ represents the eigenvector centrality value in the characteristic vector; and *a*_*ij*_ represents the contribution of node *i* to the status of node *j*. *C*(*e*)_*i*_ represents the eigenvector centrality of node *i*.

Regarding PSC, previous researches have mostly used whether executives have government service experience to determine if a company possesses PSC [64]. After conducting preliminary statistics on the resumes of executives in the sample of M&A events, we found that the majority of management teams have political connections. Using a binary variable wouldn’t capture this diversity. Thus, we adopt the method proposed by Deng et al. (2014) and measure PSC by the number of executives with political connections [92]. The formula for calculating PSC is as follows:

$$PCS\left(v_{i}\right)=\sum_{1}^{n}p_{k}$$ (4)

Here, *n* is the total number of management personnel, and when executive *k* has previously held positions in government institutions, *p*_*k*_ = 1; otherwise, *p*_*k*_ = 0.

In terms of FSC, previous studies measured whether executives had a background in financial institutions to assess a company’s FSC. When we examined the resumes of executives in listed media enterprises, we found that most enterprises had executives with experience in financial institutions. Therefore, using a binary virtual variable is not appropriate. After considering data availability and operability, we measure FSC using the number of management personnel with financial affiliations. The formula for calculating FSC is as follows:

$$FCS\left(v_{i}\right)=\sum_{1}^{n}f_{k}$$ (5)

Here, *n* is the total number of management personnel, and when management personnel *k* have previously held positions in financial institutions, *f*_*k*_ = 1; otherwise, *f*_*k*_ = 0.

#### 3.2.3 Regression model

To test the validity of the theoretical hypotheses in this study and analyze the impact of executives’ social capital on M&A performance in listed media enterprises, we used CSC, PSC, and FSC as explanatory variables for executives’ social capital (SC), and used short-term M&A performance (SP) and long-term M&A performance (LP) as dependent variables for M&A performance (P), and constructed Model (1) and Model (2). The regression model controlled such variables as the financial characteristics, governance structure, and M&A transaction features of the enterprises (Table 1). To avoid endogeneity, financial characteristic control variables were lagged by one period.

$$\begin{array}{ll} {\text{P}}_{\text{t}}\;= & \alpha_{0}+\alpha_{1}{\text{SC}}_{\text{t}}+\alpha_{2}Size_{t-1}+\alpha_{3}Tobin\text{'}Q_{t-1}+\alpha_{4}Liquidity_{t-1}\\ & +\alpha_{5}ROA_{t-1}+\alpha_{6}Lev_{t-1}+\alpha_{7}Top1_{t}+\alpha_{8}Indep_{t}+\alpha_{9}Equity_{t}\\ & +\alpha_{10}Gov_{t}+\alpha_{11}Duality_{t}+\alpha_{12}Property_{t}+\alpha_{13}Exp_{t}\\ & +\alpha_{14}Relevance_{t}+\alpha_{15}Paytype_{t}+\sum \alpha_{i}Year_{i}+\varepsilon \;(1) \end{array}$$

$$\begin{array}{ll} {\text{P}}_{\text{t}}\;= & \alpha_{0}+\alpha_{1}{\text{SC}}_{\text{t}}+\alpha_{2}{\text{SC}}_{\text{t}}\times Property_{t}+\alpha_{3}Size_{t-1}+\alpha_{4}Tobin\text{'}Q_{t-1}\\ & +\alpha_{5}Liquidity_{t-1}+\alpha_{6}ROA_{t-1}+\alpha_{7}Lev_{t-1}+\alpha_{8}Top1_{t}+\alpha_{9}Indep_{t}\\ & +\alpha_{10}Equity_{t}+\alpha_{11}Gov_{t}+\alpha_{12}Duality_{t}+\alpha_{13}Property_{t}+\\ & \alpha_{14}Exp_{t}+\alpha_{15}Relevance_{t}+\alpha_{16}Paytype_{t}+\sum \alpha_{i}Year_{i}+\varepsilon \;(2) \end{array}$$

**Table 1:** Control variables information.

Variable name	Variable name	Variable symbol	Variable description
Financial characteristics control variable	Company Size	CS	Natural logarithm of total assets
	Investment Opportunities	IO	Company’s Tobins ’ Q
	Cash Flow	CF	(cash flow—the sum of monetary funds and trading financial assets)/total assets
	Profitability	ROA	Net Profit/Total Assets
	Corporate Leverage	CL	Assets and liabilities
Governance structure control variable	Ownership Concentration	OC	The shareholding ratio of the largest shareholder
	the proportion of independent directors	ID	Number of Independent Directors/Number of Board Members
	Dual Job	DJ	Whether the chairman and the general manager are concurrently held at the same time, if it is 1, otherwise it is 0
	Management shareholding ratio	MS	Total management shareholding/Total number of shares
	Property Rights	PR	State-owned property is 1, and private property is 0
Transaction Features control variable	M&A Size	MASize	Amount of M&A transactions/Total assets at the end of the year before M&A
	Related M&A	Related	Dummy variable, 1 for related M&A and 0 for unrelated M&A
	Payment Method	Pay	Dummy variables, cash payout, is 1, the stock payout is 2, the mixed payout is 3

## 4. Results

### 4.1 Descriptive statistical

Table 2 presents the descriptive statistics of various variables. The average values for SP and LP are 0.036 and -0.451, respectively. Comparing the maximum and minimum values, it can be observed that there is relatively little variation in short-term performance among enterprises, while there is a wider range in long-term performance. The mean value for CSC is 6.29, indicating some variation in corporate relationships among listed media enterprises. The large difference between the median and maximum value of CSC suggests that a few enterprises hold higher-level positions in the relational network. The mean values for PSC and FSC are 2.738 and 1.138, respectively, and they are close to the median values, indicating that although most listed media enterprises have established both types of social capital, there is still some variability.

**Table 2:** Descriptive statistics.

Panel A: Continuous variables				
Symbol	Mean	Minimum	Maximum	Standard deviation
SP	0.036	-0.383	0.406	0.144
LP	-0.451	-4.387	3.089	0.871
CSC	6.29	0	15	3.775
PSC	2.738	0	19	3.191
FSC	1.138	0	5	1.071
CS	21.651	18.219	23.553	0.877
IO	2.467	0.255	10.032	1.571
CF	0.067	-0.312	0.291	0.088
ROA	6.949	-6.738	31.141	4.648
CL	28.942	1.778	90.475	17.097
OC	34.839	7.089	75.779	16.516
ID	37.4	25	60	0.053
MS	13.091	0	63.75	17.033
MASize	42.831	0.023	1345.237	123.611
Panel B: Categorical variables				
Label	Variable Classification		Quantity	Proportion
DJ	1		92	62%
	0		57	38%
PR	1		57	38%
	0		92	62%
Related	1		57	38%
	0		92	62%
Pay	Cash		94	63%
	Stock		7	5%
	Mixed		48	32%

### 4.2 Regression results

We conducted regression analyses based on data related to M&A events involving listed media enterprises to examine the impact of executives’ social capital on M&A value creation. Table 3 presents the regression results.

**Table 3:** SC and M&A performance regression results.

	Model (1) SP			Model (1) LP		
	(1)	(2)	(3)	(1)	(2)	(3)
CSC	0.1608***			-0.0283		
	(2.80)			(-0.11)		
PSC		0.0058**			0.0230***	
		(2.03)			(4.04)	
FSC			0.0153***			-0.0348**
			(4.77)			(-2.51)
Control	Yes	Yes	Yes	Yes	Yes	Yes
Cons	0.2415**	0.6560	0.2064*	0.1026	0.7028	0.1199
	(2.11)	(1.52)	(1.82)	(0.21)	(1.41)	(0.25)
Adj. R^2^	0.1441	0.3473	0.1482	0.2825	0.2879	0.2846

Note:

*, **, *** indicate significant at 10%, 5%, 1% levels, respectively

According to the regression results in Model (1), it is evident that the coefficients for both CSC, FSC, and PSC are significantly positive. These results suggest that when executives of media enterprises possess more social capital, either in the form of CSC, PSC, or FSC, short-term post-M&A performance tends to be better. Moving to Model (2), it is observed that the coefficient for PSC is significantly positive, indicating that when executives possess more social capital in terms of political connections, enterprises tend to achieve better long-term M&A performance. However, the coefficient of CSC does not show statistical significance, and the coefficient of FSC is significantly negative. In summary, these results suggest that executives’ social capital, especially in the form of political connections, can have a significant impact on both short-term and long-term post-M&A performance in listed media enterprises. However, CSC seems not to play a statistically significant role in shaping long-term post-M&A performance, and FSC has different impacts on short-term and long-term M&A performance.

Will the property rights of enterprises moderate the impact of social capital on M&A performance? We further explored this and presented the research results in Table 4. We found that property rights significantly moderate the impact of social capital on short-term M&A performance. Specifically, state ownership enhances the positive effect of corporate social capital (CSC) but weakens the positive effects of political social capital (PSC) and financial social capital (FSC). Concerning the long-term M&A performance, state ownership enhances the positive influence of PSC on long-term performance while mitigating the negative impact of FSC.

**Table 4:** The moderating effect of property right on SC and M&A performance.

	Model (2) SP			Model (2) LP		
	(1)	(2)	(3)	(1)	(2)	(3)
CSC	0.2129**			-0.1843		
	(2.28)			(-0.63)		
PSC		0.0392***			-0.0263	
		(9.43)			(-1.35)	
FSC			0.0227***			-0.0858***
			(5.28)			(-3.37)
CSC*State	0.8922***			0.5055		
	(4.15)			(1.02)		
PSC*State		-0.0457***			0.0875***	
		(-9.90)			(4.20)	
FSC*State			-0.0169***			0.0842*
			(-2.59)			(1.76)
Control	Yes	Yes	Yes	Yes	Yes	Yes
Cons	0.7416*	0.7198*	0.2277**	0.1201	1.1496	-0.3248
	(1.77)	(1.69)	(2.01)	(0.25)	(0.91)	(-0.28)
Adj. R^2^	0.3532	0.3815	0.1498	0.2825	0.2966	0.2611

Note:

*, **, *** indicate significant at 10%, 5%, 1% levels, respectively

### 4.3 Robustness detection

To ensure the robustness of our regression results, we adopted the approach of using proxy variables for robustness testing, as suggested by previous studies such as Lv (2018) [93]. Specifically, we calculated the BHAR for listed media enterprises within the [−10,10] time window around the M&A announcement as a proxy variable for SP. Additionally, we used the CAR calculated based on a matched sample combination approach as a proxy variable for LP. Tables 5 and 6 present the results of the robustness tests. The results of the robustness tests align with those presented in Tables 3 and 4, confirming the robustness of the conclusions drawn in this study.

**Table 5:** SC and M&A performance robustness test results.

	Model (1) SP			Model (1) LP		
	(1)	(2)	(3)	(1)	(2)	(3)
CSC	0.2108***			0.1159		
	(3.04)			(0.68)		
PSC		0.0078**			0.0093**	
		(2.07)			(2.46)	
FSC			0.0162***			-0.0202**
			(4.19)			(-2.18)
Control	Yes	Yes	Yes	Yes	Yes	Yes
Cons	0.3747***	0.7495	0.3272**	1.2790***	1.4992***	1.2416***
	(2.71)	(1.34)	(2.39)	(3.89)	(4.39)	(3.81)
Adj. R^2^	0.1386	0.1371	0.1409	0.3107	0.3125	0.3121

Note:

*, **, *** indicate significant at 10%, 5%, 1% levels, respectively

**Table 6:** Moderating effect of property right on SC and M&A performance robustness test results.

	Model (2) SP			Model (2) LP		
	(1)	(2)	(3)	(1)	(2)	(3)
CSC	0.6745***			0.0800		
	(5.51)			(0.40)		
PSC		0.0442***			-0.0140	
		(7.78)			(-1.64)	
FSC			0.0239***			-0.0647***
			(4.60)			(-4.37)
CSC*State	0.7936***			0.1168		
	(2.81)			(0.35)		
PSC*State		-0.0521***			0.0267***	
		(-8.14)			(3.05)	
FSC*State			-0.0175**			0.0475*
			(-2.22)			(1.78)
Control	Yes	Yes	Yes	Yes	Yes	Yes
Cons	1.0324*	0.9342*	0.3492**	1.2806***	1.4350***	1.2312**
	(1.88)	(1.66)	(2.54)	(3.89)	(4.21)	(2.16)
Adj. R^2^	0.3462	0.3607	0.1402	0.3105	0.3152	0.2765

Note:

*, **, *** indicate significant at 10%, 5%, 1% levels, respectively

### 4.4 Further study

In the empirical analysis above, we examined the impact of three types of social capital on short-term and long-term M&A performance. We confirmed that heterogeneous resources and the signals they send to the market can influence M&A performance during the transformation and upgrading of Chinese media enterprises. However, according to resource dependence and resource bricolage theories, resources such as social capital owned by corporate executives have to undergo a series of transformations before ultimately improving performance. Based on this, we hypothesized that the impact of executive social capital on M&A performance is gradually exhibited over dynamic development. Therefore, we further conducted a dynamic longitudinal examination of the impact of the three types of social capital on M&A performance over time to verify our hypothesis. We calculated the performance values at 6 months and 12 months post-M&A as the dependent variables for Model (2) for regression testing (results shown in Table 7).

**Table 7:** Dynamic examination results of the impact of executives’ social capital on M&A performance.

	Model (2) LP								
	T = 6			T = 12			T = 24		
CSC	-0.262			-0.559			-0.028		
	(-1.13)			(-1.58)			(-0.11)		
PSC		-0.007			-0.004			0.023***	
		(-1.20)			(-0.89)			-4.04	
FSC			-0.0063			-0.048***			-0.035**
			(-0.48)			(-2.63)			(-2.51)
Control	Yes	Yes	Yes	Yes	Yes	Yes	Yes	Yes	Yes
Cons	1.217***	1.095**	1.268***	2.717***	1.330***	2.733***	0.103	0.703	0.120
	-2.62	-2.26	-2.75	-3.98	-3.3	-4.03	-0.21	-1.41	-0.25
Adj R^2^	0.156	0.1562	0.155	0.1902	0.1993	0.1919	0.2825	0.2879	0.2846

Note:

*, **, *** indicate significant at 10%, 5%, 1% levels, respectively

When T = 6, the regression coefficients of CSC, PSC, and FSC are all not significant; when T = 12, the regression coefficients of CSC and PSC remain not significant, but the regression coefficient of FSC is significantly negative; when T = 24, the regression coefficient of CSC is not significant, the regression coefficient of PSC becomes significantly positive, and the regression coefficient of FSC remains negative. This result indicates that the three types of executives’ social capital begin to have a significant impact on M&A performance at different times. The earliest social capital to significantly impact M&A performance is financial social capital, which shows a negative impact on performance starting 6 months post-M&A, and this negative impact persists up to the 24th month. Political social capital, on the other hand, begins to show a positive impact 12 months post-M&A. This result shows that the influence of executives’ social capital on M&A performance evolves dynamically over time.

## 5. Discussion

### (1) Impact of corporate social capital

Based on the regression results, CSC has a significant positive impact on short-term M&A performance but does not significantly impact long-term performance. Therefore, Hypothesis 1 is only partially supported. For countries where formal institutions are not fully developed (such as China), this study confirms that resource-rich and interdependent networks can provide enterprises with sustained competitive advantages [94, 95]. In a highly competitive environment, enterprises within alliances are stronger than standalone ones or those with few alliances [96]. As outlined by social exchange theory, resource exchange and interdependence are the foundations of relationships [97, 98]. Although previous research shows that executives with greater network capabilities can effectively search for, scan, discover, and identify individuals or organizations holding resources, find common interests and possibilities for resource exchange, and then develop diversified connections with these individuals or organizations to further establish strong and beneficial alliances [99], our findings suggest that resource exchange theory can be used to explain the enhancement of M&A performance through CSC only during the initial stage of M&A for media enterprises undergoing transformation and upgrading.

In the early stages of M&A, according to social resource exchange theory, when the executives of media enterprises possess more CSC, the enterprise has more information dissemination channels, stronger informational advantages, and the ability to find high-quality acquisition targets, thereby reducing information search costs [100]. Additionally, with more CSC, executives are more likely to acquire experiential knowledge and even internal information within the corporate network, facilitating the smooth progress of the M&A [101]. Therefore, after the M&A announcement, it is more likely to be recognized by the capital market, resulting in an improvement in short-term M&A performance. However, in the later stages of M&A, our research results indicate that more CSC neither significantly promotes M&A performance nor leads to a decline in corporate performance due to the defense effect, as found by El-Khatib et al. (2015) [80]. This means that during the transformation and upgrading process, executives do not use corporate social network as an information channel to harm shareholders’ interests through M&A, neither do they leverage corporate social network as a social resource exchange channel in the M&A process. The reasons for this are as follows. (1) the need for immediate performance improvement during transformation and upgrading. The transformation and upgrading of Chinese media enterprises began when the media market was severely hit by the Internet-based content industry, causing a substantial decline in media enterprises’ revenue [102]. In this context, the fundamental purpose of M&A is to quickly improve performance [103], giving executives less opportunity to use their social network as an information channel to harm shareholders’ interests. (2) high homogeneity of exchangeable resources due to multiple concurrent positions in the same industry. This study measures CSC using executives’ concurrent positions in different organizations. Most executives of media enterprises hold positions in other enterprises in the same region, resulting in highly homogeneous exchangeable social resources. Therefore, CSC does not have a significant impact on long-term M&A performance.

Additionally, the CSC of executives in state-owned media enterprises enhances short-term M&A performance more effectively than in private enterprises. In China’s listed media enterprises, executives of state-owned enterprises (SOEs) are subject to stricter position management and supervision, resulting in fewer opportunities to hold positions in other organizations [104]. This prevents the phenomenon observed in previous research where executives’ excessive concurrent positions lead to increased workloads, ultimately reducing supervision and management efficiency [81, 105], but this is not the case for private enterprise executives, as they are not subject to the same institutional constraints. Moreover, SOE executives have closer ties with government departments, enhancing their prestige compared to private enterprise executives [106], which helps them gain more social resources through their social networks [107].

### (2) Impact of political social capital

PSC has a positive impact on both short-term and long-term M&A performance, thereby validating Hypothesis 2. Although existing research found that in Chinese enterprises’ overseas M&A and M&As in the energy sector, political social capital can have a significantly negative impact on M&A performance due to the government’s irrational control [108], that is not the case for Chinese media enterprises undergoing transformation and upgrading. For these enterprises, political social capital has a significantly positive impact on both their short-term and long-term M&A performance, which aligns with the resource-based view. Due to the unique nature of the Chinese media industry, critical resources are controlled by the government, and most M&As require government approval [67]. Therefore, executives’ PSC is important in obtaining M&A resources and overcoming industry and regional barriers. Additionally, the transformation and upgrading of Chinese media enterprises began at a time when the industry suffered a significant impact from the internet industry [109, 110]. In response, the Chinese government initiated a media integration strategy [111, 112], aimed at enhancing the competitiveness of media enterprises through technological and organizational integration, and M&A is a crucial means of implementing this strategy [3].

According to the signaling theory, enterprises with rich PSC, due to their closer ties with the government, send a positive signal to the market after M&A events announcement that they are more likely to receive government support, which boosts investor confidence. The social exchange theory suggests that human relations are based on rational choice [113] and constitute a resource-exchange relationship—an exchange of goods, both material and non-material, such as the symbols of approval or prestige. Executives with government work experience have greater influence due to their prestige and are more likely to gain others’ trust and useful resources [114], thereby enhancing short-term M&A performance. In addition, under policy guidance, media enterprises can have access to tax incentives and government subsidies during their transformation and upgrading [115]. More PSC means easier access to those resources, which helps alleviate enterprises’ financial pressure after M&A, thus promoting long-term M&A performance. Additionally, the impact of PSC on long-term M&A performance evolves dynamically. In the 6 months and even 12 months after the M&A, PSC’s impact on long-term M&A performance is not significant. However, 24 months post-M&A, PSC starts to show a significantly positive impact on long-term M&A performance. This is due to the delayed effect of tax incentives and government subsidies [116]. When tax incentives and government subsidies begin to play a role in the company’s finances, the market will respond accordingly.

Besides, the property rights attribute has a moderating effect on the impact of PSC. In the short term after M&A, the impact of PSC on the performance of SOE is significantly smaller than that on private enterprises. In the current institutional environment in China, the executives of state-owned media enterprises are mainly appointed by government departments and typically enjoy higher political levels, and therefore they often undertake more political objectives and social responsibilities in M&A activities [117]. Concerns about the "exploitative hand of the government" lead to a lack of market confidence in M&A activities involving too many non-economic factors [118], thereby weakening the positive effect of PSC on the short-term M&A performance of state-owned media enterprises. However, in the long run, although state-owned media enterprises undertake more political objectives and social responsibilities in M&A, they receive more tax incentives, government subsidies, loan support, and policy preferences [119], which alleviate their financial burden during transformation and upgrading [120]. Therefore, PSC has a greater positive effect on the long-term performance of M&A in state-owned media enterprises.

During the transition of China’s economy, private enterprises face dual dilemmas: On the one hand, due to relatively poor institutional construction, the role of the government as a provider of public goods has not been effectively implemented. Market failures and dysfunctions make it difficult to effectively guarantee the signing and execution of contracts, resulting in unusually high transaction costs for economic entities, including private enterprises, which inhibit the effective improvement of resource allocation efficiency. On the other hand, due to restrictions on factors such as capital and technology, as well as policy barriers resulting from ownership discrimination, the development of private enterprises is greatly constrained [121]. Therefore, in this context, informal social network relationships emerge as important economic alternatives to formal institutions. Compared to other types of enterprises, private enterprises pay more attention to establishing and expanding relational networks and are more willing to invest more resources in this to get support and protection that they can’t obtain from formal institutions [90]. For private media enterprises, when executives establish political factions to gain rent-seeking interest, they also incur corresponding rent-seeking costs [122], hence the impact of PSC is not as significant as it is for state-owned enterprises.

### (3) Impact of financial social capital

FSC has a negative impact on short-term M&A performance but has a positive effect on long-term M&A performance. Therefore, hypothesis 3 is not supported. The findings of this study are only partially consistent with those of Chaudhry et al. (2022) [123], as both studies found that FSC can effectively promote short-term M&A performance, while the impact on long-term M&A performance is inconsistent. This study found that network theory can only explain the impact of FSC on performance in the short term after M&A, while the impact of FSC in the longer term after M&A does not align with network theory. From the perspective of network theory, financial institutions often act as intermediaries for information, serving as channels for executives to access information flow, which helps alleviate information asymmetry and reduce M&A premiums [124]. At the same time, the relationship between executives and financial institutions, as an informal institution, effectively alleviates the financing constraints of enterprises [86]. Sufficient FSC gives enterprises greater access to external funds, thereby alleviating their financial pressure during M&A, and improving the performance of the enterprise. Therefore, executives with abundant FSC in the early stages of M&A send positive signals to the market, leading to an increase in stock prices upon M&A event announcement, thereby enhancing M&A performance.

However, one year after the M&A event announcement, FSC begins to exert a negative influence on M&A performance. From an agency perspective, the reduction of financing constraints brought about by abundant FSC generates sufficient cash flow, encouraging executives to engage in non-value-maximizing M&A activities due to agency issues [125, 126]. Additionally, more FSC typically indicates closer relationships between executives and financial institution personnel, which although guarantee easier access to loans and financing to some extent, may also lead to lax supervision by financial institutions [127, 128], making executives or major shareholders more prone to engage in M&A activities for personal interests or out of excessive optimism [129]. At the same time, due to the urgent need for transformation and upgrading, the targets of M&A activities for media enterprises are mostly technology companies such as gaming, digital media, and digital marketing enterprises. According to the annual financial reports released by media enterprises after M&A, their goodwill growth is significant, indicating that the premium for M&A by media enterprises is significantly higher than in other industries in China [130]. Sufficient cash flow combined with high M&A premiums often signals to the market the existence of agency issues in M&A activities [131], leading to a decline in M&A performance. Within media enterprises, executives with abundant FSC are more likely to maximize personal interests through M&A during the transformation and upgrading process, leading to agency problems.

Compared to private media enterprises, FSC has a greater positive impact on the short-term performance of state-owned media enterprises and a smaller negative impact on their long-term performance. In China’s institutional environment, most financial resources are concentrated in SOEs [132]. Although private enterprises can appoint individuals with a background in financial institutions as executives to alleviate financing constraints through social networking with financial institutions, financing restrictions still exist [133]. Therefore, compared to SOEs, FSC does not bring about much performance improvement for private enterprises in the short term after M&A. However, SOEs have to undergo stricter review systems for M&A events, which can effectively prevent M&A activities caused by excessive investment and other agency issues resulting from abundant cash flow [134].

## 6. Conclusion and recommendations

### 6.1 Conclusion

As digital technology and media integration continue to advance, China’s media industry is undergoing profound transformation and has successfully upgraded itself into a pillar industry of the national economy. During this process, media enterprises actively seek to achieve their strategic goals of transformation and upgrading by acquiring new resources, technologies, and market shares through M&A. For enterprises in other emerging economies aiming to implement transformation and upgrading amid the digital economic revolution, the successful transformation and upgrading of Chinese media Enterprises serve as a valuable reference.

Starting from the perspective of resource dependence theory, we categorized the executives’ social capital into CSC, PSC, and FSC. We chose 145 M&A events involving listed media enterprises that occurred between 2008 and 2017 as the sample. Through empirical analysis, we examined the impact of heterogeneous social capital on M&A performance, as well as the moderating effect of property rights within this context. The research results indicated the following: (1) CSC significantly enhances short-term M&A performance but has no significant effect on long-term performance; (2) PSC positively influences both short-term and long-term M&A performance. State-owned media enterprises may gain relatively fewer benefits from PSC in the short term after M&A, but they can accrue more significant benefits in the long term. (3) FSC does not affect short-term M&A performance but exerts a negative impact on long-term performance. This negative effect is even more pronounced in state-owned enterprises.

### 6.2 Recommendations

This study found that in the process of transformation and upgrading, corporate executives’ social capital plays a pivotal role in driving the implementation of M&A strategies and enhancing performance. To optimize a company’s social capital and improve M&A performance, we propose the following strategies:

#### (1) Establish a dedicated corporate relationship management team and expand corporate social capital through social media

While corporate executives build relationships and acquire social capital, their influence may be diminished due to the multiple concurrent positions they hold in different organizations. Therefore, enterprises can establish a management team dedicated to building social capital. This team can develop broad relational networks through social activities such as industry roundtable meetings and strategic partnership workshops, thereby acquiring more business opportunities and strengthening the enterprise’s influence within the industry. Simultaneously, enterprises can employ social media strategies to expand their corporate social capital network. For instance, they can regularly publish content on industry trends, company achievements, and profound insights through social media to engage potential business partners and industry experts. Establishing online social platforms to encourage interaction and collaboration among professionals is another effective approach. Furthermore, enterprises can cultivate close relationships with suppliers, customers, and business partners. By investing in long-term cooperation, they can collectively foster growth and address market challenges. Establishing a sustainable partner ecosystem enables business growth and innovation.

#### (2) Actively engage in government projects and establish a political risk management mechanism

In industries undergoing transformation and upgrading, such as the media industry, which are considered pillars of the national economy, the government is likely to provide support for corporate M&A activities. Consequently, during periods of transformation and upgrading, especially for private enterprises, it is crucial to engage in policy analysis and government relationship management effectively. Actively seek advice from government departments and maintain contact to gain in-depth insights into policy dynamics. Actively participating in government projects that align with the enterprise’s strategic direction maximizes access to government resources and support. When state-owned enterprises participate in government projects, they should formulate clear government cooperation strategies to ensure these collaborations align with their corporate strategic objectives. Additionally, considering the potential rent-seeking costs associated with political social capital, enterprises should establish a political risk management mechanism to assess and address the potential impacts of political factors on their business. Regular political risk assessments and crisis response plans should be implemented to ensure business continuity and stable government relations.

#### (3) Build diversified financial partnerships, and strengthen risk detection, and compliance management

When enterprises pursue transformation and upgrading through M&A, financial social capital can provide ample cash flow but may also give rise to issues such as agency problems and relaxed external regulation. Therefore, enterprises, especially state-owned enterprises, should establish strong relationships with various financial partners, including banks, venture capitalists, private equity investors, and angel investors to maintain their financial social capital. Ensuring the diversification of financial relationships mitigates risks associated with financial social capital. Additionally, establish a risk monitoring mechanism to track the compliance and potential risks of financial relationships. Build dedicated internal audit and compliance teams to promptly identify and address potential risks and implement appropriate corrective measures.

### 6.3 Limitations and prospects

This study has the following limitations: (1) This study constructs corporate networks only with executives’ relationships, but does not include shareholder networks when measuring social capital. (2) This study only takes the property rights attributes of enterprises as an observational variable for the institutional environment but does not incorporate other observational variables such as the level of market development, legal regulations, etc., into the research model.

Nonetheless, these research limitations do not affect the accuracy of the conclusions but inspire further research. In future studies, we will take enterprises from other industries or regions as samples to further examine the robustness and universality of the research conclusions.

## Supporting information

S1 Dataset The data set used in this article for discussion and analysis. (ZIP)
